# Development and characterization of an HPV18 detection kit using two novel HPV18 type-specific monoclonal antibodies

**DOI:** 10.1186/s13000-018-0727-7

**Published:** 2018-08-17

**Authors:** Yao Zhang, Ye He, Ling Li, Shutian Liang, Mei Yan, Dongyan Ren, Zengmin Yang, Wenli Zhao, Luyan Miao, Haijiang Zhang, Yongjiang Liu

**Affiliations:** Beijing Health Guard Biotechnology Co., Ltd., Unit 201 & 202, Block 2, Longsheng Industrial Park, 7 Rongchang East Street, Beijing Economic and Technological Development Area, Daxing District, Beijing, 100176 People’s Republic of China

**Keywords:** ELISA kit, HPV, Monoclonal antibody, Vaccine

## Abstract

**Background:**

HPV 18 is one of the most prevalent oncogenic types, only second to HPV 16, and included in the licensed vaccines on the market. In this study, we describe the production and characterization of a panel of monoclonal antibodies (mAb) to HPV18.

**Methods:**

The immunocompetence of 1B1 and 4C2 mAbs for HPV L1 protein was evaluated by SDS-PAGE analysis, neutralization assays, affinity identification, and ELISA. The 1B1 and 4C2 genes were sequenced and analyzed. Finally, the detection kit with the two mAbs was assessed for linearity, repeatability and specificity.

**Results:**

Both mAbs specifically recognized HPV18 L1 and virus-like particles (VLPs). These mAbs are conformation-neutralizing antibodies that have high affinity and type specificity. Based on these characteristics of these mAbs, we developed an ELISA kit for specifically detecting HPV 18 antigen. We showed that this kit displayed good linearity, repeatability and sensitivity for detecting HPV18 L1 pentamer and HPV18 VLP.

**Conclusions:**

We characterized two monoclonal neutralizing antibodies for HPV L1 protein, and developed an ELISA kit for specifically detecting HPV 18 antigen. This newly developed kit can be used to monitor the potency of HPV vaccines throughout the entire production process as well as preliminary analysis of HPV18 infections.

## Background

Human papillomaviruses (HPVs) are a group of non-enveloped viruses with a double-stranded DNA [1, 2], which mainly infect epithelial cells and can further induce a variety of benign and malignant hyperplasia [3]. The high-risk, oncogenic HPV types are related to invasive cervical cancer and other genital carcinomas; whereas the low-risk HPVs cause anogenital warts [4, 5]. The high-risk types include HPV types 16, 18, 31, 33, 35, 39, 45, 91, 52, 56, 58, 59, 68, 69, 73, and 82, among which HPV type 16 (HPV16) and HPV type 18 (HPV18) together are responsible for approximately 70% of cervical cancer cases [6]. Because the diseases caused by HPV infections are a serious harm to human health, the development of safe and effective preventive or therapeutic vaccines is of great significance [7].

There is an early-gene and a late-gene region within the genome of HPV virus. The early genes E1-E7 play roles in viral life cycle and pathogenesis, whereas the late genes L1 and L2 code for the viral capsid proteins L1 and L2 respectively [8–11]. L1 is the major capsid protein, and 72 copies of L1 pentamers make up the viral envelope; L2, the minor capsid protein, sits in the center of the L1 pentamers [8, 11, 12]. Numerous studies have confirmed that HPV-L1 protein is the major target protein for HPV vaccine: without HPV-L2 protein, the HPV-L1 protein expressed in vitro can self-assemble into virus-like particles (VLPs) that mimic the structure of the native virus [13, 14]. The L1-VLPs retain the vast majority neutralizing epitopes of the natural virus, and can induce high titers of neutralizing antibodies [15, 16]. Therefore, L1-VLPs of different types of HPV are the major component of the HPV vaccines currently available or during development.

Three prophylactic vaccines against HPV are currently available on the market. Cervarix™ (GSK) is a bivalent vaccine designed to prevent high-risk HPV type 16 and 18 infections [17], which has recently been approved by China FDA. Gardasil^®^ (Merck) — a quadrivalent vaccine — targets two oncogenic types (HPV16 and HPV18) and two types that cause genital warts (HPV6 and HPV11) [1, 18]. Merck has developed a nonavalent vaccine targeting five additional high-risk HPV types (HPV 31/33/45/52/58) to the HPV types 6/11/16/18 contained in Gardasil^®^ [17], and was licensed by U.S. FDA in December 2014 [18]. The nonavalent HPV vaccine Gardasil^®^ 9 appears to be safe and effective in preventing persistent infection and precancerous lesions associated with HPV types 16/18/31/33/45/52/58, as well as genital warts related to HPV types 6 and 11 [19].

It is crucial to monitor the quality of products of every step during the development and production of vaccines. For HPV vaccines, tests for the purpose of quality control include HPV type identification experiment, determination of antigen content, in vitro and in vivo potency assays. These tests utilize antibody binding and competition, making quantifications and comparisons of samples easy to be completed and adapted to other platforms [4, 20, 21]. The antibodies used in such tests are often HPV type-specific, and need to reflect the biological properties of the original potency and neutralization assays [20, 22].

During our research and development of a nonavalent HPV vaccine (valence include 6/11/16/18/31/33/45/52/58, same as Gardasil^®^ 9 by Merck), we produced hybridoma cell lines that produce type-specific HPV18 neutralizing mAbs, and assembled an ELISA detection kit using two of the mAbs. This kit, which can rapidly identify and quantify HPV18 L1-VLPs in HPV vaccines, is crucial for vaccine quality control. More importantly, this kit can be used in preliminary clinical analysis of HPV18 infections, and the mAbs can be applied in the development of more sophisticated immunohistochemistry systems.

## Methods

Experimental mice were handled by licensed laboratory animal practitioners in all procedures, and were injected intraperitoneally with nembutal sodium for euthanasia at the end of the experiments.

### Production of hybridomas and generation of mAbs

BALB/c mice were given two subcutaneous injections sequentially, with one being 10 μg HPV18 L1-VLP protein mixed with complete Freund’s adjuvant, and the other one being the antigen mixed with Freund’s incomplete adjuvant [3]. 15 μg HPV18 L1-VLP protein in normal saline was given intraperitoneally as a final boost 3 days prior to fusion [20, 23]. Mice spleen cells collected following the standard procedure were fused with SP2/0 myeloma cells [Mouse Hybridoma (Sp 2/0), IMM010.51.2] (ATCC, USA) in a 10:1 ratio in 500 g/L PEG4000 [3]. Fused hybridomas were isolated through hypoxanthine-aminopterin-thymidine (HAT) medium selection, and supernatants were screened using a sandwich enzyme-linked immunosorbent assay (ELISA) for reactivity [20]. Using the limiting dilution method, positive wells were cloned, and the hybridoma cell lines were cultured and passaged in DMEM culture medium containing 10% fetal bovine serum. The stable hybridoma cell lines secreting monoclonal antibody were obtained after 10 generations of well and stable growth. Mature BALB/c mice were inoculated with hybridoma cells. Supernatant of ascites collected from these mice were purified on Protein G affinity columns (Upstate Biotechnology, Lake Placid, NY, USA) for HPV18 L1-VLP mAbs. The purity of the purified mAbs was confirmed using SDS-PAGE.

The supernatants of the wells containing the hybridoma cells were tested using indirect ELISA: 96-well microtiter plates were coated with HPV18 L1-VLP (200 ng per well). The serum from immunized mice was used as a positive control; the supernatants of cultures with no cell growth as well as the serum from normal mice were the negative controls. The wells were subsequently incubated with horseradish peroxidase-conjugated (HRP-conjugated) anti-mouse IgG (100 μl per well) at a ratio of 1:2000. The absorption of the supernatants at 450 nm was measured in an ELISA reader.

### Characterization of monoclonal antibodies generated against human papillomavirus type 18 Virions

#### Identification of antibody isotypes

Using the antibodies against various mice IgG isotypes, the IgG isotypes of the antibodies produced by the hybridoma cells were identified by the indirect ELISA method.

#### The reactivity of antibodies and HPV18L1-VLP by indirect ELISA

The antibodies against HPV18 L1-VLP were detected using the indirect ELISA method: 96-well microtiter plates were coated with HPV18 L1-VLP (200 ng/well), and mAbs were added subsequently at concentrations from 1 μg/ml to 0.04 μg/ml. The binding strength of each purified mAbs to the HPV18 L1-VLP was determined by ELISA.

#### Identification of antibody specificity

The identification of antibody specificity was achieved by means of the indirect ELISA method using denatured L1-VLPs and mAbs. Briefly, native L1-VLPs were denatured by incubation in denaturation buffer [0.2 M sodium carbonate, pH 10.6, 10 mM dithiothreitol (DTT)] for 30 min and boiling for 5 min. The 96-well microtiter plates were coated with L1-VLP (200 ng per well) overnight at 4 °C. After drying, the plates were blocked with 2% BSA (300 μl/well) for 2 h, and incubated with antibodies (100 μl at 0.3 μg/ml) for 1 h at room temperature. HRP-conjugated goat anti-mouse IgG (1:4000 in assay diluent) was added to the dried plates and incubated for 1 h at room temperature. The plates were washed with PBS 5 times prior to the addition of the chromogenic substrate solution (100 μl/well). The reaction was stopped by the addition of the termination solution (100 μl/well) after 10-min incubation in the dark, and the absorbance was measured at 450 nm.

#### Neutralization assays against mAbs

Neutralization assays with HPV18 pseudovirus (PsV18) were performed in 293FT cells as described previously [1, 22].

All mAbs were diluted to a nominal concentration of 200 μg/ml, and each mAb was further diluted by a 4-fold serial dilution. 50 μl PsV18 (at a pertinent concentration) was incubated with equal volume of mAbs for 1 h at 4 °C; PsV18 in PBS was used as the negative control. Subsequently, the antibody-pseudovirus mixture was incubated with 293FT cells (Invitrogen, USA). Seventy-two hours post seeding, the cells were harvested, and subjected to a flow cytometer to measure the fluorescence. The fluorescence inhibition rate was calculated as follows:

$$ fluorescence\ inhibition\ rate=\left(1-\frac{experimental\ group}{blank\ control\ group}\right)\times 100\% $$

A 50 and 90% inhibited concentration (IC_50_ and IC_90_) was determined for neutralization titer of the monoclonal antibody against PsV18.

#### Competition experiment of neutralizing serum against antibody in vitro by ELISA

HPV18 L1-VLP antigen (10 ng/well) was incubated in 96-well plates overnight at 4 °C. The plates were washed and blocked with 2% BSA for 2 h at room temperature. Subsequently, 100 μl international standard (Anti-human papillomavirus[HPV] 18 serum, NIBSC) at 0.5 IU/ml concentration and equal volume of HRP-conjugated antibodies (0.3 μg/ml) were added to each well, with PBS as a negative control. Plates were incubated for 1.5 h at room temperature. The reaction was stopped, developed, and the absorbance was measured at 450 nm. The calculation of inhibition rate followed the equation below:

$$ inhibition\ rate=\left(1-\frac{OD_{experimental\ group}}{OD_{control\ group}}\right)\times 100\% $$

#### The binding affinity of mAbs 1B1 and 4C2

In order to reflect the degree of binding of the antibody to the HPV18 L1-VLPs, the antigen-antibody binding and dissociation were analyzed using a BIACORE3000 biosensor (GE Healthcare); the binding constants *K*_*a*_, the dissociation constant *K*_*d*_ and the binding affinity *K*_*D*_ were calculated.

According to the manufacturer’s instructions, the HPV18 L1-VLPs was diluted to a concentration of 40 μg/ml (with acetic acid/sodium acetate buffer, pH 5.5), and coupled to the chip with the coupling level of 4000 RU.

The antibodies were respectively diluted to seven concentrations in PBS, ranging from 0.3125 to 20 nM. After sampling for 60 s, binding for 60 s and dissociating for 500 s, the chip was regenerated using acetic acid/sodium acetate buffer, pH 5.0. According to the manufacturer’s instructions, the binding kinetics data were analyzed using Biacore 3000 Evaluation program.

#### Sequencing of 1B1 and 4C2 genes

Two hybridoma cell lines (1B1 and 4C2) were harvested, and total mRNA was extracted, from which cDNA was synthesized by RT-PCR. The cDNA was amplified with high fidelity using variable region universal primers, and the PCR products were inserted into T carrier separately to determine their DNA sequences. The DNA sequences were translated into amino acid sequences that were aligned and analyzed subsequently.

### Detection kit for HPV18 L1-VLP

#### Linearity and repeatability of the detection kit

The 1B1/4C2 antibody pairing experiment using the sandwich ELISA method was performed to confirm the coating antibody and the detecting antibody respectively. The antigens were diluted to concentrations of 10μg/ml, 3μg/ml, lug/ml, 0.3μg/ml and 0.1μg/ml respectively, and the ELISA experiments were performed as described to identify batch-to-batch and inter-batch variations. The assays were repeated 10 times for each sample.

#### Assembly of the detection kit

The coating antibody 4C2 was diluted to a concentration of 10 μg/ml in 0.05 M sodium carbonate buffer, pH 9.6. 96-well plates were incubated overnight at 4 °C with 100 μl/well coating antibody. The plates were washed with PBST twice and blocked with 3% BSA (100 μl/well) at 37 °C. Two hours post seeding, the plates were washed with PBS, and protected with 10% aqueous sucrose solutions for 1 h at room temperature. The dried plates were sealed in vacuum aluminum foil bags and stored at 4 °C.

The HRP-conjugated antibodies were named 1B1-HRP. 96-well plates were incubated for 1.5 h at 37 °C with 100 μl/well HPV18 L1-VLPs. After washing, 100 μl 1B1-HRP at 0.5 μg/ ml was added to each well, and allowed to react with the HPV18 L1-VLPs for 1 h at 37 °C. The dried plate was developed for 10 min at 37 °C prior to the addition of the termination solution (50 μl/well). The absorbance was measured at 450 nm.

#### The specificity test of detection kit

The specificity test of the Detection Kit was performed as follows: native L1-VLPs were denatured by incubation in denaturation buffer (0.2 M sodium carbonate, pH 10.6, 10 mM DTT) for 30 min at room temperature and boiling for 5 min. Each well of a 96-well plate was used to test 3 μg HPV18 L1-VLPs in PBS.

## Results

### Production of hybridomas and generation of mAbs

A total number of 10 mAbs against HPV18 L1-VLPs were developed from mouse hybridoma cells following the standard method, two of which were used to construct the HPV18 Detection Kit. To avoid any confusion, we only present data regarding to these two mAbs.

**Fig. 1:**
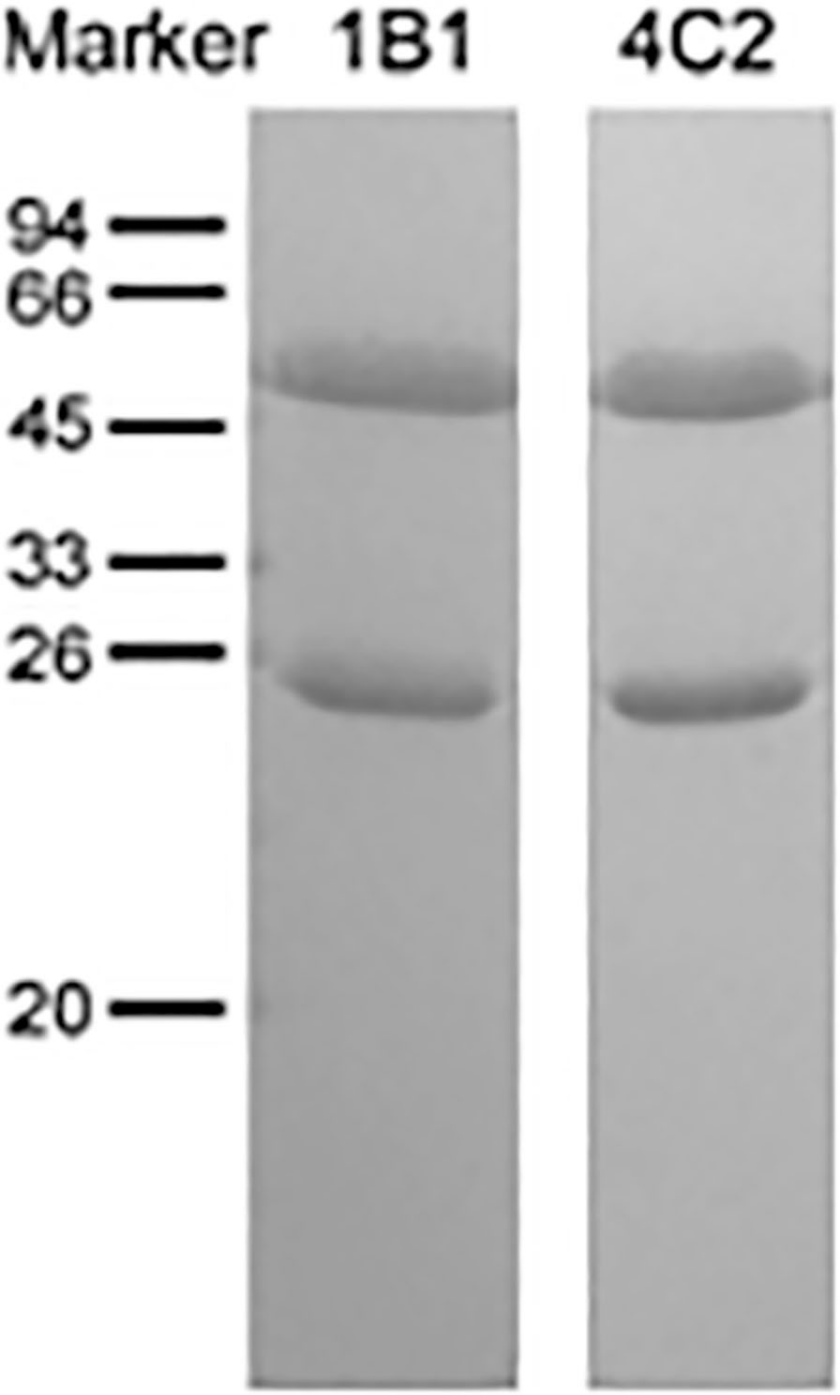
SDS-PAGE analysis of purified mAbs. The mAbs of 1B1 (lanes 1) and 4C2 (lanes 2) were purified and analyzed as described. The two bands with a molecular weight of 25 K and 50 K respectively in each lane correspond to the light- and heavy-chain of that mAb

Both of the purified mAbs 1B1 and 4C2 were analyzed by SDS-PAGE electrophoresis, where two bands were observed in each sample lane. The molecular weights of the two bands were 25 kDa and 50 kDa respectively, corresponding to the light- and heavy-chain of the antibody respectively. The purity of both mAbs was more than 95% (Fig. 1).

### Characterization of monoclonal antibodies generated against human papillomavirus type 18 Virions

The antibody isotypes of all mAbs obtained were identified by indirect ELISA in early stage screenings, and the results suggested that both 1B1 and 4C2 belonged to the IgG1 isotype. Once the isotypes were confirmed, the mAbs were subjected to further characterizations.

**Fig. 2:**
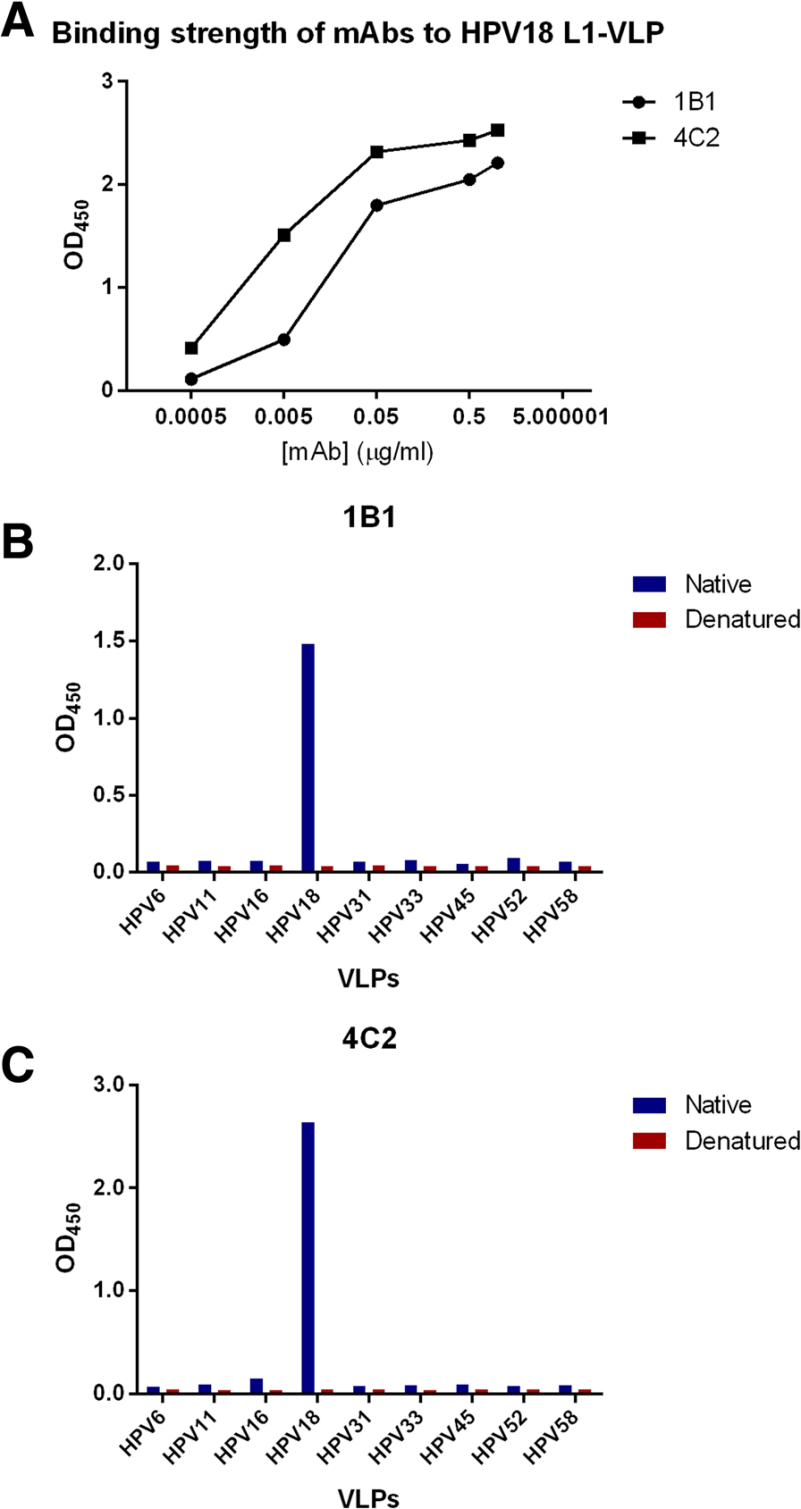
Characterization of the mAbs against HPV18 L1-VLP. **a** The binding strength of mAbs 1B1 and 4C2 to HPV18 L1-VLPs. OD_450_ is the optical density at 450 nm; the concentrations of the mAbs are given in μg/ml. The reaction curves were sharply increased at the turning point of 0.05 μg/ml. The results indicate that both 1B1 and 4C2 mAbs have similar binding capabilities to HPV18 L1-VLPs; **b** and **c** Specificities of mAbs 1B1 and 4C2 to native or denatured HPV L1-VLPs. The optical density at 450 nm (OD_450_) of mAbs 1B1, 4C2 mAbs were plotted on the y-axis against various genotypes of native or denatured VLPs on the x-axis. The bar graph was prepared using Prism GraphPad 6.0. All mAb samples were adjusted to an initial concentration of 0.3 μg/ml. All values are the average of multiple holes. L is the native protein, and D is the denatured protein

The reactivity of these mAbs towards HPV18 L1-VLPs was tested at different concentrations using the indirect ELISA method. The binding strength of each mAb to HPV18 L1-VLPs was shown in Fig. 2a. Both mAbs could bind to HPV18 L1-VLPs at all concentrations used in this experiment, and the signals for both mAbs were greater than 1 when the concentrations of the mAbs reached 0.05 μg/ml.

Subsequently, the specificities of 1B1 and 4C2 to HPV L1-VLPs within nine types of HPV (6/11/16/18/31/33/45/52/58) were measured by indirect ELISA. Both native and denatured L1-VLPs were used to determine whether they were conformational epitope-specific antibodies. Our results showed that both 1B1 and 4C2 mAbs reacted with native HPV18 L1-VLPs, and had a signal of > 1; whereas the OD_450_ values of other reactions were < 0.1 (Fig. 2b). These results suggested that neither 1B1 nor 4C2 had cross reactivities with other types of HPV, thus they are HPV18 type-specific antibodies. Comparing with the group using native HPV18 L1-VLPs, a significant decrease in the OD_450_ value of samples reacted with denatured HPV18 L1-VLPs would suggest these mAbs were conformational epitope-specific. As a conclusion, both of the mAbs were specific clones to HPV18 L1-VLPs.

The neutralization activities of the mAbs were tested following a well-characterized in vitro pseudovirus-cell neutralization assay. The neutralization titers of the mAbs against PsV18 were determined at IC_50_ and IC_90_ (Table 1). The IC_50_ of 1B1 and 4C2 were 0.039 μg/ml and 10.00 μg/ml respectively; and the IC_90_ of each mAbs were 0.156 μg/ml and 40.140 μg/ml respectively. These data demonstrated that both mAbs had a certain degree of inhibition reactions, and thus neutralization activities. The lower the IC_50_, the higher the neutralizing activity towards HPV18 L1-VLPs the mAb had.

**Table 1:** Characteristics of the HPV18-VLP mAbs

MAb	Isotype^a^	Epitope recognized	Inhibitory concentration (μg/ml) of mAbs against HPV-PsV infection		Inhibition rate^c^
			IC_50_	IC_90_	
1B1	IgG1	C^b^	0.039	0.043	48.63%
4C2	IgG1	C	10.000	0.037	33.98%

^a^antibody isotype, classified by indirect ELISA;

^b^Conformational epitope, confirmed by indirect ELISA;

^c^tested upon competition experiment of neutralizing serum against antibody in vitro by ELISA

We examined whether our mAbs were competitive with the international standard anti-HPV18 neutralizing antibody from the World Health Organization (WHO) by competitive ELISA assay. By applying the International Standard to the HPV18 L1-VLP coated plates prior to the application of our mAb, any epitopes recognized by both the International Standard and our mAb would be blocked by the International Standard, which would result in fewer mAb binding in the next step, thus a lower OD value than that of the control group. This experiment would reveal the correlation of the epitopes recognized by our mAbs with those by the International Standard – the higher the inhibition rate, the more similar the epitopes recognized by our mAb to those by the International Standard. As shown in Table 1, the inhibition rates of 1B1 and 4C2 mAbs were 48.63 and 33.98% respectively, suggesting that the epitopes recognized by 1B1 and 4C2 have a certain degree of correlation with those by the International Standard.

We also analyzed the antigen-antibody binding and dissociation kinetics using L1-VLPs of the nine HPV types (6/11/16/18/31/33/45/52/58), and calculated the binding constant *K*_*a*_, disassociation constant *K*_*d*_, and the binding affinity *K*_*D*_ for each reaction (Table 2). The *K*_*D*_ of 1B1 and 4C2 were in the nanomolar range, suggesting they are high affinity antibodies. Amongst all the reactions, the binding constant *K*_*a*_, the dissociation constant *K*_*d*_ and the affinity *K*_*D*_ of mAbs 1B1 and 4C2 were only detected towards HPV18 L1-VLPs, but not for the other types of HPV tested, suggesting that these two mAbs were HPV18 type-specific.

**Table 2:** Binding affinity of mAbs 1B1 and 4C2

mAb	HPV18			Others^b^		
	*K*_*a*_ (Ms^− 1^)^a^	*K*_*d*_ (s^− 1^)^a^	*K*_*D*_ (M)^a^	*K*_*a*_ (Ms^− 1^)	*K*_*d*_ (s^− 1^)	*K*_*D*_ (M)
1B1	9.31 × 10^5^	1.92 × 10^− 3^	2.06 × 10^−9^	N/A^c^	N/A	N/A
4C2	2.38 × 10^6^	1.78 × 10^−2^	7.45 × 10^− 9^	N/A	N/A	N/A

^a^*K*_*a*_ is the binding constants, *K*_*d*_ is the dissociation constant, *K*_*D*_ is the binding affinity;

^b^HPV L1-VLP of other types, including HPV16, HPV58, HPV6, HPV11, HPV31VLP, HPV33, HPV45, and HPV52;

^c^Not Applicable

**Fig. 3:**
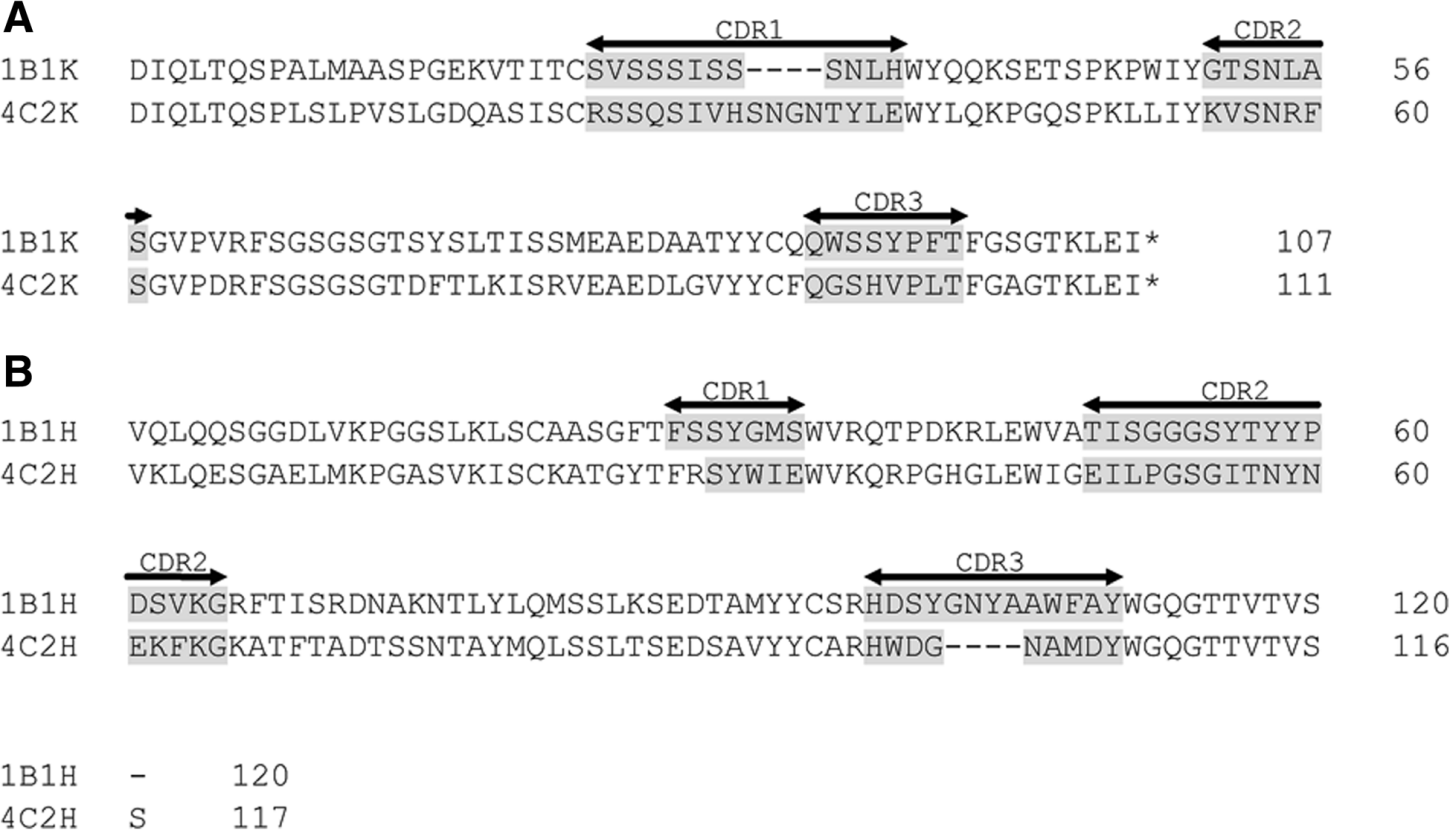
Alignments of amino acid sequences of the (**a**) light- and (**b**) heavy-chain regions of mAbs 1B1 and 4C2. Complementarity-determining regions (CDRs) are shaded gray

To identify the mAbs obtained, mAbs 1B1 and 4C2 were sequenced. Amino acid sequence alignments of the light- and heavy-chains of these two mAbs are shown in Fig. 3. The gray-shaded complementarity-determining regions (CDRs) of 1B1 and 4C2 vary in the amino acid sequences, suggesting the obtained mAbs are two unique antibodies with the same type specificity.

### Detection kit for HPV18 L1-VLP

**Fig. 4:**
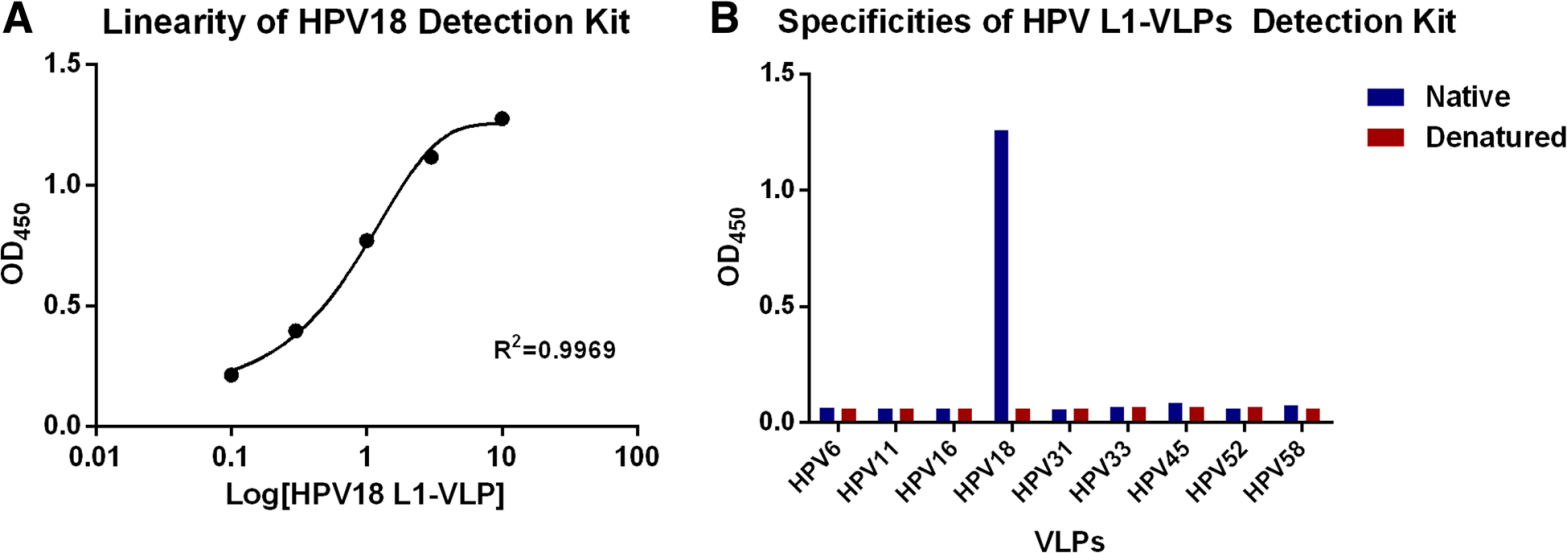
Characterization of the HPV18 L1-VLP Detection Kit developed in this study. **a** The linearity of the HPV18 L1-VLP Detection Kit. Each well was coated with 10 μg/ml of mAb 4C2, blocked with 3% BSA-PBST, and air-dried. Different concentrations (0.1, 0.3, 1.3, and 10 μg/ml) of the HPV18 L1-VLP standards were diluted in 3% BSA-PBST and tested in triplicate. The positive control was defined as being the mean of greater than 2.1times the negative control. The standard curve was sigmoidally fitted with 4 parameters; x axis is the logarithm of HPV18 L1-VLP concentrations; y axis was the optical density at 450 nm (OD_450_); **b** The specificities for native and denatured HPV L1-VLPs of the Detection Kit. The OD_450_ values of all samples tested were plotted against various genotypes of HPV L1-VLPs (both native and denatured). The bar graph was prepared using Prism GraphPad 6.0. All L1-VLPs samples were adjusted to an initial concentration of 30 μg/ml (100 μl per well). All of the values were the average of multiple samples

Using 4C2 as the coating antibody and HRP-conjugated 1B1 as the detecting antibody, the linearity range of the Detection Kit was determined. The Curve was fitted with Four Parameter Equation, and as shown in Fig. 4a, the linearity range of the Detection Kit was from 0.1 μg/ml to 10 μg/ml.

The specificity of the Detection Kit was determined using both native and denatured L1-VLPs of HPV6, 11, 16, 18, 31, 33, 45, 52, and 58. Amongst all the samples used, only native HPV18 L1-VLPs gave a positive signal (Fig. 4b). The results showed that the Detection Kit could detect native HPV18 L1-VLPs with a good sensitivity and have no cross-reactivity with HPV L1-VLPs of other types. It also suggested that the mAbs used in this kit recognized conformational epitopes on HPV18.

## Discussion

In general, when developing and selecting mAbs for a detection kit of HPV18, we seek to satisfy four criteria. First, the sequence of each mAb must be a specific sequence in the kit. Second, the mAbs should have good binding affinity towards the antigen and would not rapidly dissociate [20]. Third, two mAbs used in the same assay should be able to quickly and accurately identify the antigen than other mAbs. Last but not least, the mAbs should represent critical quality attributes of the kit, such as linearity, repeatability and affinity.

In this study, two mAbs (1B1 and 4C2) were screened from 10 mAbs expressed by hybridoma cells stably expressing type-specific neutralizing antibodies against HPV18 L1-VLPs. A few features of mAbs 1B1 and 4C2, including antibody isotype, HPV type specificity, conformational epitopes, neutralization ability, and sequences were characterized. Both 1B1 and 4C2 had good specificity towards native HPV18 L1-VLPs and showed no cross-reactivity to the L1-VLPs of the other eight HPV types tested. Compared with antibodies described in similar studies [7, 15] that have cross-neutralizing activities towards other types of HPVs, our mAbs are HPV18 type-specific and recognize conformational epitopes on HPV18 L1-VLPs.

Amongst all the HPV vaccines currently available, the nonavalent Gardasil^®^ 9 has the widest protection range. Our mAbs, which were demonstrated to have high titers only towards HPV18 L1-VLPs within the 9 HPV types analyzed (valences same as those of Gardasil^®^ 9), can be used in the specific detection of HPV18 VLPs in HPV vaccines with up to 9 valences or compounds that have HPV18 component. Furthermore, they can be applied to the detection of HPV18 infections in clinic.

This pair of mAbs was subsequently used to develop an HPV18 Detection Kit. One mAb coated on the ELISA plate would capture the L1-VLPs, whereas the other mAb with conjugated HRP was used for detection. Such sandwich ELISA approach uses two mAbs to detect and quantify HPV18, allowing the detection of not only HPV18 L1 pentamers, but also HPV18 L1-VLPs. The detection limit of the kit was 0.l μg/ml, with a linear range of 0.l – 10 μg/ml. Assays have also confirmed the kit had high specificity and sensitivity only towards native HPV18 L1-VLPs. This Detection Kit has been applied in our routine quality control tests of HPV vaccine productions, and it was also used by other organizations in relevant analyses.

This Detection Kit was designed primarily for the quality control throughout HPV vaccine production as well as preliminary detection of HPV types. We have taken this research further by developing a more sophisticated IHC system using these mAbs, aiming for a more accurate and convenient detection of HPV18 infections in tissue samples (to be reported elsewhere).

## Conclusions

In summary, high-affinity and strongly neutralizing antibodies were generated, characterized, and used to develop a sandwich ELISA kit that could identify and quantify HPV18 type-specific L1-VLP antigens within the nonavalent HPV vaccine range (HPV6/11/16/18/31/33/45/52/58). With the two mAbs we produced, the Detection Kit could detect the presence of HPV18 L1 pentamer or HPV18 L1-VLPs from up to nine different types of HPVs, thus could be widely used in quality control for clinical testing and vaccine manufacturers. This study has also provided us a guideline for the rational design of high-affinity monoclonal antibodies that could be applied to the diagnosis and treatment of HPV18 infections.

## Abbreviations

HAT: Hypoxanthine-aminopterin-thymidine
HPV: Human Papillomavirus
HRP: Horseradish peroxidase
mAb: Monoclonal antibody
PsV: Pseudovirus
VLP: Virus-like particle
